# Supplementary material for: The FlgN chaperone activates the Na+-driven engine of the Salmonella flagellar protein export apparatus
Source: Commun Biol. 2021 Mar 12;4:335. doi: 10.1038/s42003-021-01865-0 (PMC7955116; doi:10.1038/s42003-021-01865-0)
Supplement: Supplementary file 2 — Supplementary Information [file 42003_2021_1865_MOESM2_ESM.pdf]

# Supporting Information

# The FlgN chaperone activates the $Na^+$-driven engine of the *Salmonella* flagellar protein export apparatus

**Tohru Minamino, Miki Kinoshita,**

**Yusuke V. Morimoto and Keiichi Namba**

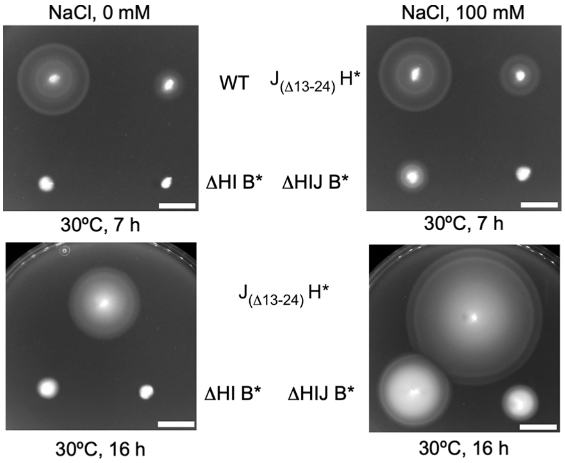


**Supplementary Fig. 1.** Motility of SJW1103 (wild-type, indicated as WT), MM104H-3 [*fliJ(Δ13–24) fliH(Δ96–97)*, indicated as $J_{(\Delta 13\text{–}24)}$ H*], MMHI0117 [*ΔfliH-fliI flhB(P28T)*, indicated as ΔHI B*] and MMHIJ0117 [*ΔfliH-fliI-fliJ flhB(P28T)*, indicated as ΔHIJ B*] in 0.35% soft agar plates in the absence (left panels) and presence (right panels) of 100 mM NaCl. Scale bar, 1.0 cm.

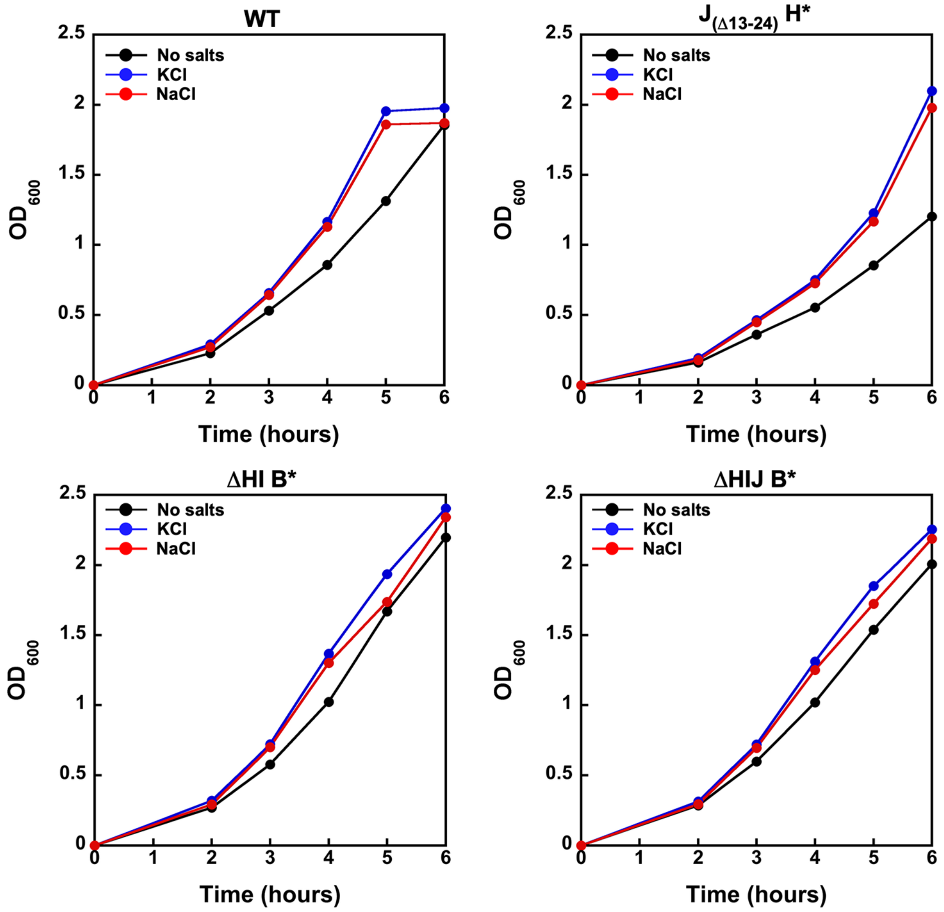


**Supplementary Fig. 2.** Effect of KCl and NaCl on the cell growth of SJW1103 (WT), MM104H-3 ($J_{(\Delta 13–24)}$ H*), MMHI0117 (ΔHI B*) and MMHIJ0117 (ΔHIJ B*). The cells were grown at 30ºC in T-broth (pH 7.5) (indicated as no salts) or T-broth (pH 7.5) containing either 100 mM KCl or 100 mM NaCl. The $OD_{600}$ was monitored. These data are the average of three independent biological replicates. The experimental errors are within 10 %.

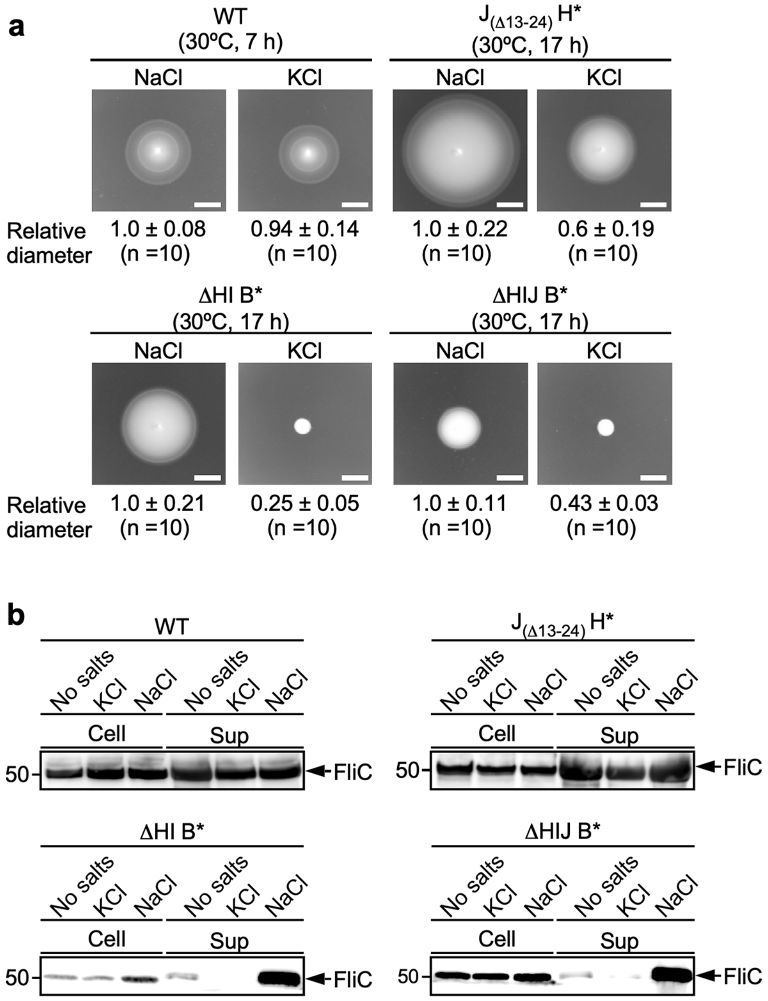


**Supplementary Fig. 3. Effect of $K^+$ on flagellar protein export. (a)** Motility of SJW1103 (WT), MM104H-3 ($J_{(\Delta 13–24)}$ H*), MMHI0117 (as ΔHI B*) and MMHIJ0117 (ΔHIJ B*) in 0.35% soft agar plates containing 100 mM NaCl or 100 mM KCl. The diameter of the motility ring of 10 colonies of each strain was measured. The average diameter of the motility ring of each strain grown in the presence of 100 mM NaCl was set to 1.0, and then the relative diameter of the motility ring of the cells grown in the presence of 100 mM KCl was calculated (mean ± SD, n = 10). Scale bar, 1.0 cm. **(b)** Effect of $K^+$ on flagellar protein export at external pH 7.5. Immunoblotting, using polyclonal anti-FliC antibody, of whole cell proteins (Cell) and culture supernatant fractions (Sup) prepared from the above strains grown exponentially at 30ºC in T-broth (pH 7.5) (indicated as no salts) or T-broth (pH 7.5) containing either 100 mM KCl or 100 mM NaCl.

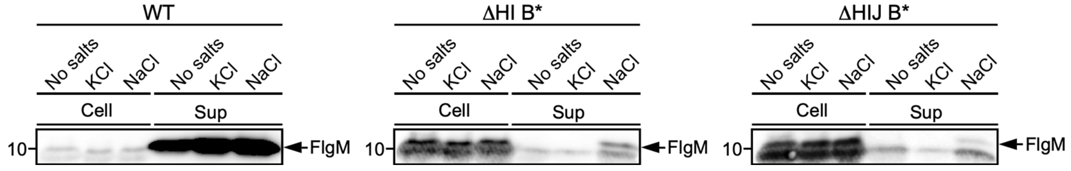


**Supplementary Fig. 4.** Effect of $Na^{+}$ and $K^{+}$ on FlgM secretion. Immunoblotting, using polyclonal anti-FlgM antibody, of whole cell proteins (Cell) and culture supernatant fractions (Sup) prepared from SJW1103 (wild-type, indicated as WT), MMHI0117 (ΔHI B*) and MMHIJ0117 (as ΔHIJ B*) grown exponentially at 30ºC in T-broth (pH 7.5) (indicated as no salts) or T-broth (pH 7.5) containing either 100 mM KCl or 100 mM NaCl.

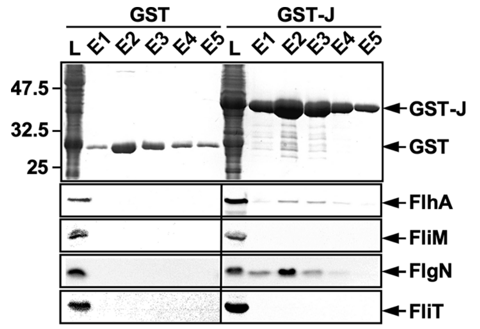


**Supplementary Fig. 5. Pull-down assays by GST affinity chromatography.** Cell lysates (indicated as L) prepared from *Salmonella* MMHI0117 (ΔHI B*) cells expressing GST or GST-FliJ (indicated as GST-J) were loaded onto a GST column. After washing with 15 ml of PBS, proteins were eluted with 5 ml of 50 mM Tris-HCl, pH 8.0, 10 mM reduced glutathione. Elution fractions were analysed by both CBB staining (1st row) and immunoblotting with anti-FlhA$_C$ (2nd row), anti-FliM (3rd row), anti-FlgN (4th row) or anti-FliT (5th row) because it has been reported that FliJ binds to FlhA$_C$, FliM, FlgN and FliT *in vitro*[1–5].

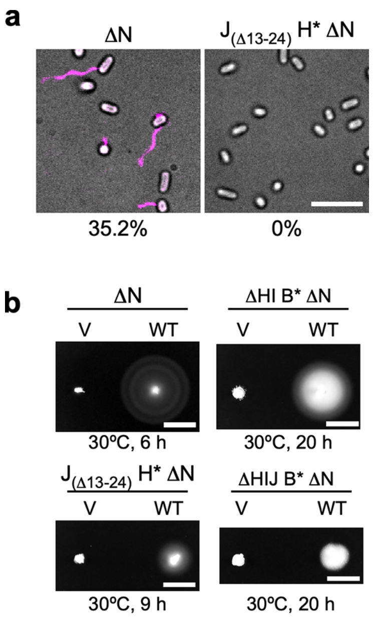


**Supplementary Fig. 6.** Effect of FlgN deletion on flagellar filament formation. **(a)** Fluorescent images of MM9001 (ΔN) and MM9003 ($J_{(\Delta 13-24)}$ H* ΔN). The flagellar filaments were labeled with Alexa Fluor 594. The fluorescence images of the filaments labeled with Alexa Fluor 594 (magenta) were merged with the bright field images of the cell bodies. About 35.2% of the MM9001 cells produced a single flagellar filament, but the MM9003 cells produced no filaments. Scale bar, 5.0 µm. **(b)** Motility of MM9001, MM9002 (ΔHI B* ΔN), MM9003 or MM9004 (ΔHIJ B* ΔN) harbouring pTrc99A (V) or pMMGN140 (FlgN) in soft agar. Scale bar, 1.0 cm.

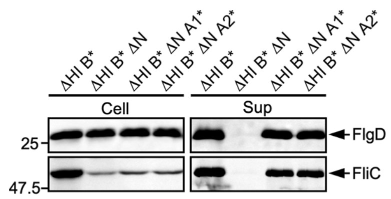


**Supplementary Fig. 7.** Effect of the *flhA(D456V)* (A1*) or *flhA(T490M)* (A2*) mutation on flagellar protein export by the ΔHI B* ΔN mutant cells. Immunoblotting, using polyclonal anti-FlgD (1st row) or anti-FliC antibody (2nd row), of whole cell proteins (Cells) and culture supernatant fractions (Sup) prepared from the MMHI0117(ΔHI B*), MM9002(ΔHI B* ΔN), HMM001 (ΔHI B* ΔN A1*) and HMM002 (ΔHI B* ΔN A2*).

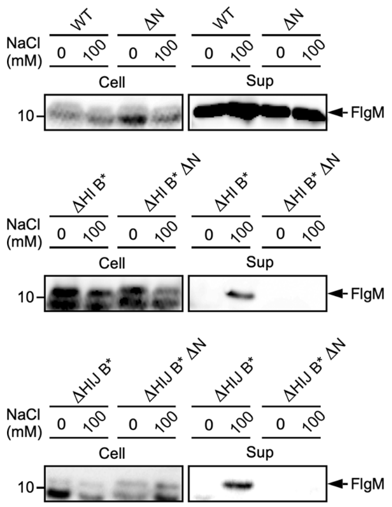


**Supplementary Fig. 8.** Effect of FlgN deletion on FlgM secretion. Immunoblotting, using polyclonal anti-FlgM antibody, of whole cell proteins (Cell) and culture supernatant fractions (Sup) prepared from SJW1103 (WT, indicated as WT), MM9001 (ΔN), MMHI0117 (ΔHI B*), MM9002 (ΔHI B* ΔN), MMHIJ0117 (ΔHIJ B*) and MM9004 (ΔHIJ B* ΔN) grown exponentially at 30ºC in T-broth (pH 7.5) with or without 100 mM NaCl.

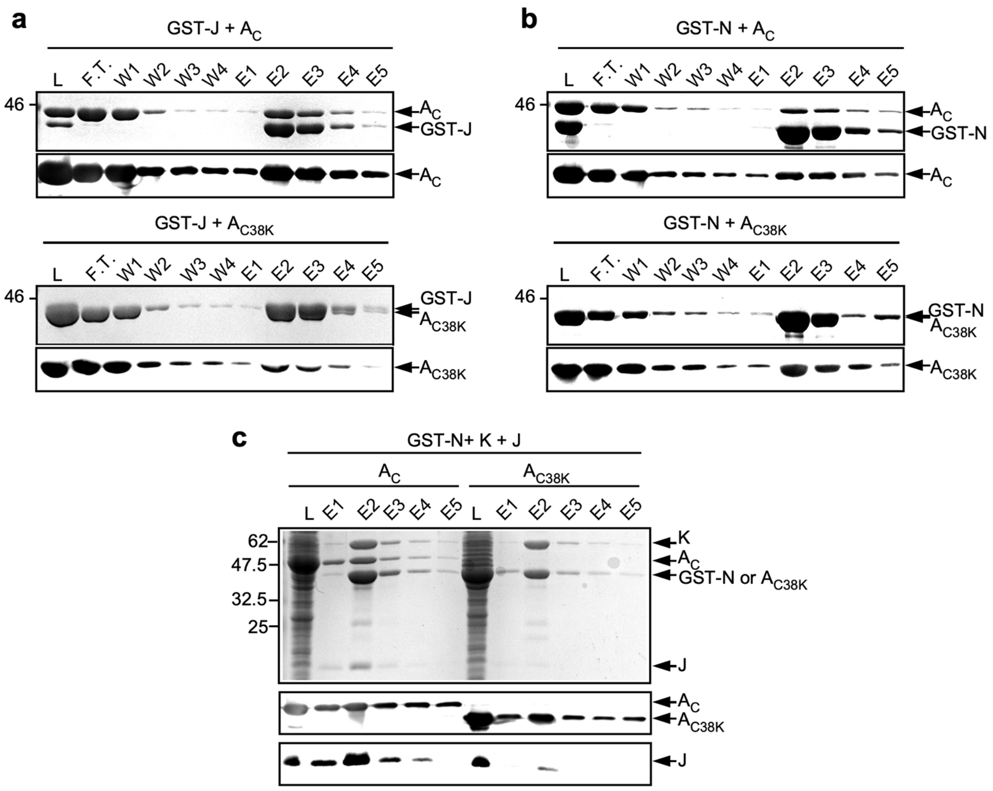


**Supplementary Fig. 9. Interactions of FlhA$_C$ with FliJ and FlgN. (a, b)** Effect of deletion of residues 328–351 of FlhA$_L$ on interactions of FlhA$_C$ with **(a)** FliJ and **(b)** FlgN. Purified His-FlhA$_C$ (indicated as A$_C$) or His-FlhA$_{C38K}$ lacking residues 328–351 of FlhA$_L$ (indicated as A$_{C38K}$) was mixed with purified GST-FliJ (indicated as GST-J) or GST-FlgN (indicated as GST-N), followed by overnight dialysis against PBS at 4ºC. Each mixture (L) was loaded onto a GST column. After washing with 10 ml PBS, proteins were eluted with 10 mM reduced glutathione. Flow through fraction (F.T.), wash fractions (W), and elution fractions (E) were subjected to SDS-PAGE, followed by both CBB staining (upper panels) and immunoblotting with polyclonal anti-FlhA$_C$ antibody (lower panels). **(c)** Effect of deletion of residues 328–351 of FlhA$_C$ on the FlgN–FliJ interaction. Pull-down assays by GST affinity chromatography. Cell lysates prepared from *Salmonella* SJW1368 (Δ*flhDC-cheW*) cells expressing either GST-FlgN (indicated as GST-N) was mixed with purified FlgK (indicated as K), purified His-FliJ (indicated as J) and the soluble fraction from *E. coli* BL21(DE3) Star cells producing either His-FlhA$_C$ (indicated as A$_C$) or HisFlhA$_C$ lacking residues 328–351 of FlhA$_L$ (indicated as A$_{C38K}$), and then each mixture (indicates as L) was loaded onto a GST column. After extensive washing, proteins were eluted with a buffer containing 10 mM reduced glutathione. Elution fractions were analyzed by CBB staining (1st row) and immunoblotting with polyclonal anti-FlhA$_C$ (2nd row) or anti-FliJ (3rd row).

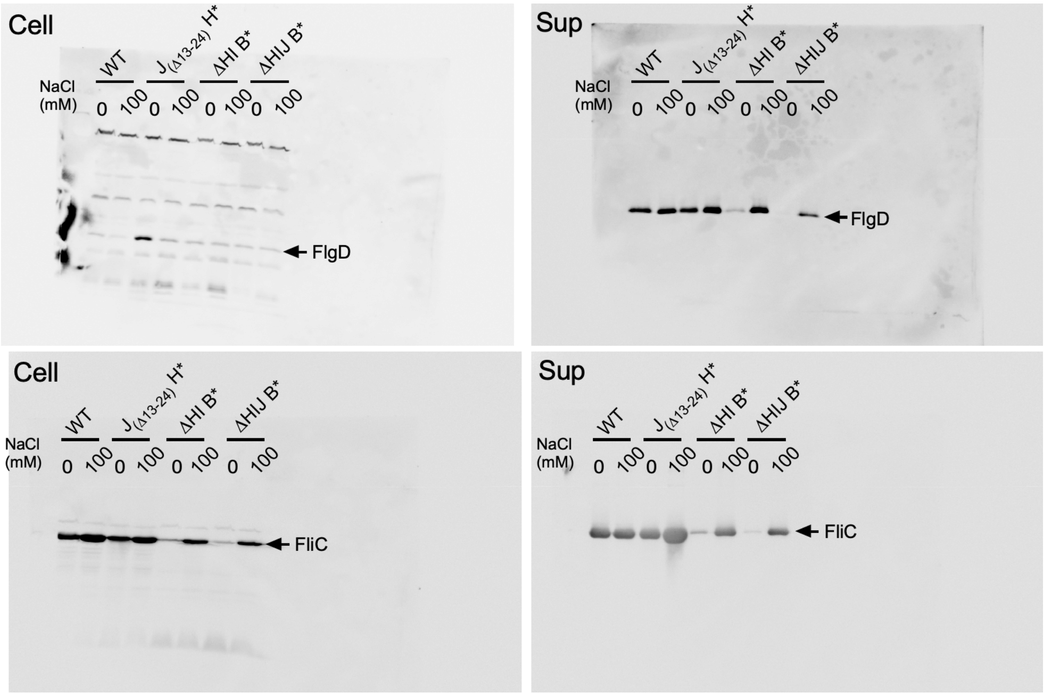


**Supplementary Fig. 10. Original immunoblots shown in Figure 1b.**

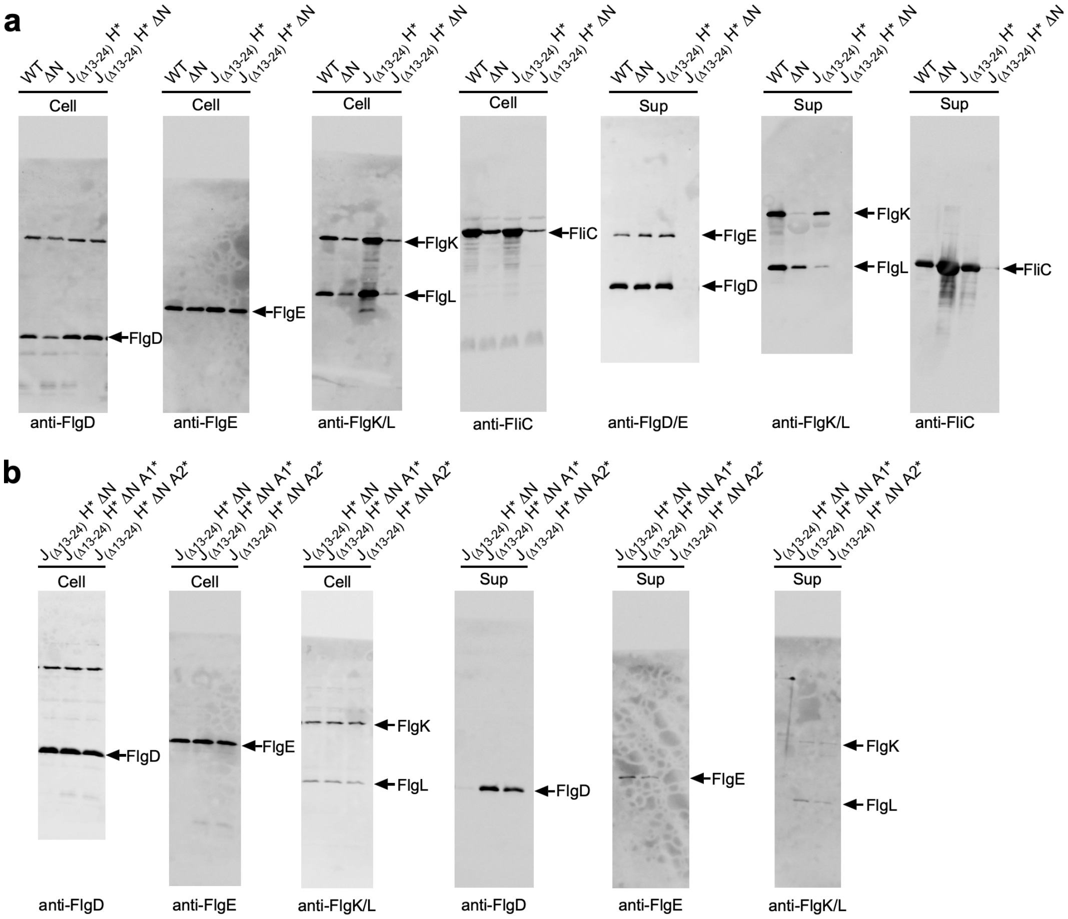


**Supplementary Fig. 11. Original immunoblots shown in (a) Figure 3b and (b) 3e.**

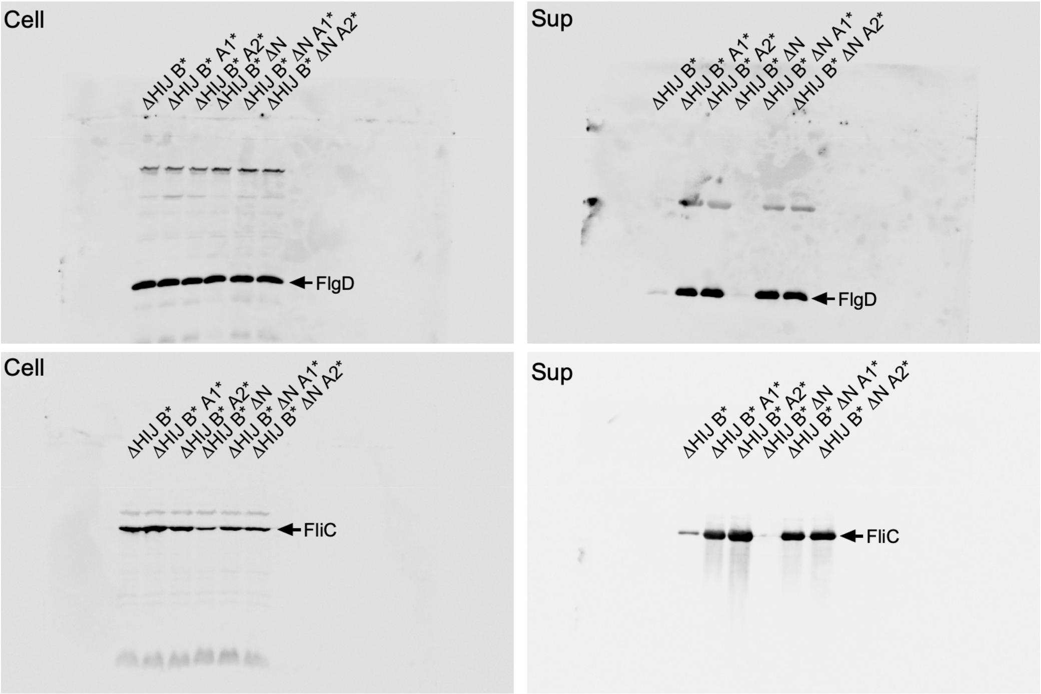


**Supplementary Fig. 12. Original immunoblots shown in Figure 4b.**

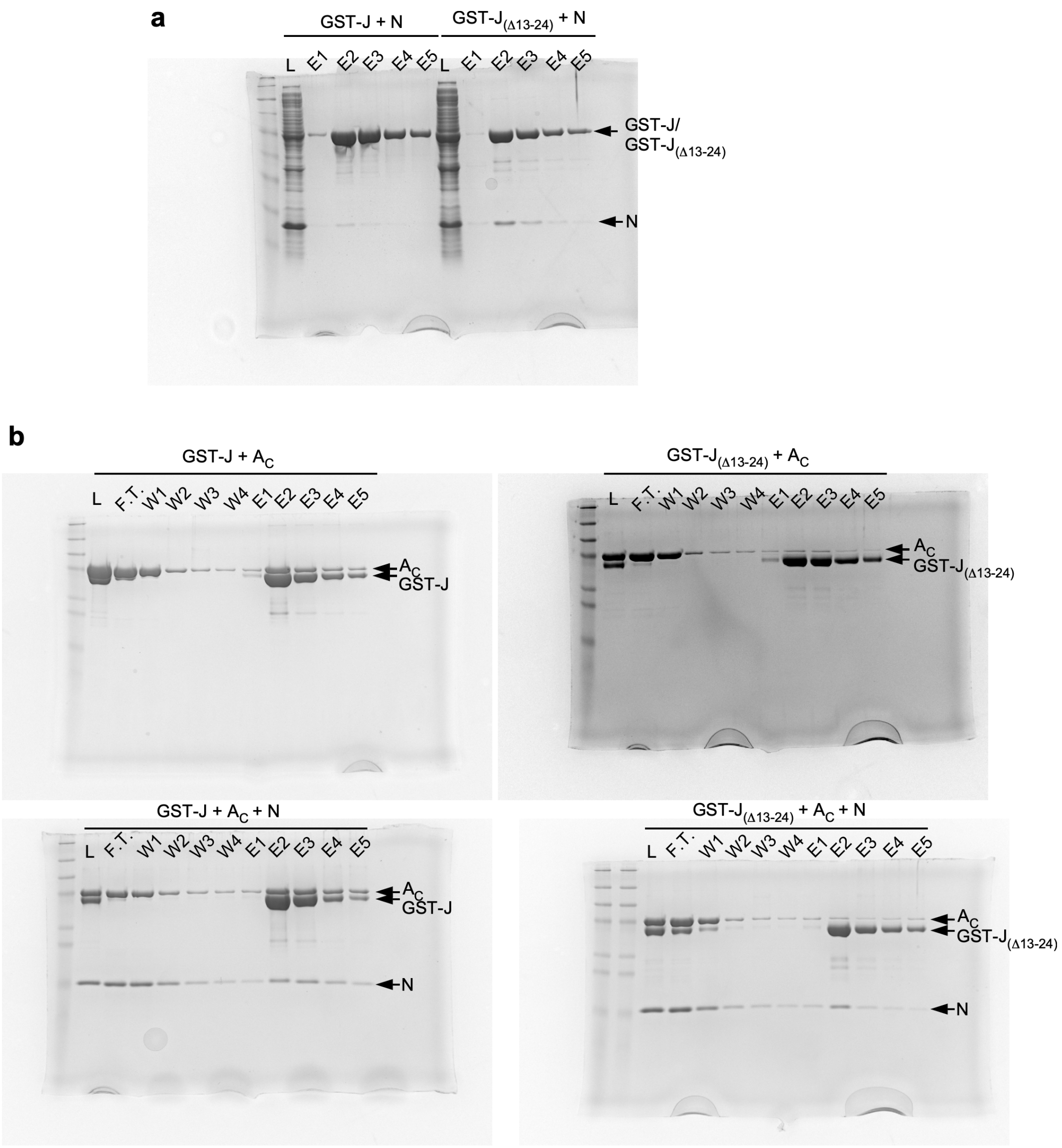


**Supplementary Fig. 13. Original CBB-stained gels shown in (a) Figure 5a and (b) 5b.**

**Supplementary Table 1. Effect of $Na^+$ ions on average number and length of flagellar filaments produced by the WT and $J_{(\Delta 13-24)}$ H* cells**

| Strain | NaCl (mM) | Fraction of flagellated cells (%) | Average number of flagella in flagellated cell (mean ± SD) | Average length of filament (μm) (mean ± SD) |
|---|---|---|---|---|
| SJW1103 (WT) | 0 | 100 (n = 152) | 2.7 ± 1.1 (n = 152) | 11.3 ± 2.1 (n = 50) |
| | 100 | 100 (n = 153) | 3.3 ± 1.5 (n = 153) | 12.9 ± 2.5 (n = 50) |
| MM104H-3 ($J_{(\Delta 13-24)}$ H*) | 0 | 61.5 (n = 192) | 1.3 ± 0.5 (n = 118) | 7.0 ± 2.9 (n = 50) |
| | 100 | 87.3 (n = 166) | 2.0 ± 1.0 (n = 145) | 10.7 ± 3.1 (n = 50) |

**Supplementary Table 2. Effects of *flhA(D456V)* (A1*) and *flhA(T490M)* (A2*) mutations in FlhA and FlgN deletion (ΔN) on average filament number and length in flagellated ΔHIJ B* cells**

| Strain | Fraction of flagellated cells (%) | Average filament number in flagellated cell (mean ± SD) | Average filament length (μm) (mean ± SD) |
|---|---|---|---|
| MMHI0117 (ΔHI B*) | 78.2 (n = 340) | 1.6 ± 0.7 (n = 266) | 7.8 ± 2.5 (n = 50) |
| MMHIJ0117 (ΔHIJ B*) | 13.5 (n = 423) | 1.1 ± 0.2 (n = 57) | 5.1 ± 2.2 (n = 50) |
| MMHIJ0117-2 (ΔHIJ B* A1*) | 95.3 (n = 359) | 1.9 ± 0.8 (n = 342) | 5.7 ± 1.5 (n = 50) |
| MMHIJ0117-3 (ΔHIJ B* A2*) | 99.2 (n = 376) | 2.1 ± 0.9 (n = 373) | 5.3 ± 1.5 (n = 50) |
| MM9002 (ΔHI B*ΔN) | 0 (n = 266) | - | - |
| MM9004 (ΔHIJ B*ΔN) | 0 (n = 329) | - | - |
| MM9004-2 (ΔHIJ B* A1* ΔN) | 20.8 (n = 355) | 1.0 ± 0.2 (n = 74) | - |
| MM9004-3 (ΔHIJ B* A2* ΔN) | 8.5 (n = 377) | 1.0 ± 0.0 (n = 32) | - |

**Supplementary Table 3. Plasmids and strains used in this study**

| **Strains/ Plasmids** | **Relevant characteristics** | **Source or reference** |
|---|---|---|
| ***E. coli*** | | |
| BL21 (DE3) Star | Over-expression of proteins | Novagen |
| | | |
| ***S. enterica* serovar Typhimurium** | | |
| SJW1103 | Wild type for motility and chemotaxis | 6 |
| SJW1368 | *ΔcheW–flhD* | 7 |
| MM104H-3 | *fliJ(Δ13–24) fliH(Δ96–97)* | 5 |
| MM9001 | *ΔflgN::tetRA* | 8 |
| MM9002 | *ΔfliH-fliI flhB(P28T) ΔflgN::tetRA* | 8 |
| MMHI0117 | *ΔfliH-fliI flhB(P28T)* | 16 |
| MMHI0117-2 | *ΔfliH-fliI flhB(P28T) flhA(D456V)* | 9 |
| MMHI0117-3 | *ΔfliH-fliI flhB(P28T) flhA(T490M)* | 9 |
| MMHIJ0117 | *ΔfliH-fliI-fliJ flhB(P28T)* | 5 |
| HMM001 | *ΔfliH-fliI flhB(P28T) ΔflgN::tetRA flhA(D456V)* | 8 |
| HMM002 | *ΔfliH-fliI flhB(P28T) ΔflgN::tetRA flhA(T490M)* | 8 |
| MM9003 | *fliJ(Δ13–24) fliH(Δ96–97) ΔflgN::tetRA* | This study |
| MM9003-2 | *fliJ(Δ13–24) fliH(Δ96–97) ΔflgN::tetRA flhA(D456V)* | This study |
| MM9003-3 | *fliJ(Δ13–24) fliH(Δ96–97) ΔflgN::tetRA flhA(T490M)* | This study |
| MMHIJ0117-2 | *ΔfliH-fliI-fliJ flhB(P28T) flhA(D456V)* | This study |
| MMHIJ0117-3 | *ΔfliH-fliI-fliJ flhB(P28T) flhA(T490M)* | This study |
| MM9004 | *ΔfliH-fliI-fliJ flhB(P28T) ΔflgN::tetRA* | This study |
| MM9004-2 | *ΔfliH-fliI-fliJ flhB(P28T) ΔflgN::tetRA flhA(D456V)* | This study |
| MM9004-3 | *ΔfliH-fliI-fliJ flhB(P28T) ΔflgN::tetRA flhA(T490M)* | This study |
| | | |
| **Plasmids** | | |
| pTrc99AFF4 | Expression vector | 10 |
| pGEX-6p-1 | Expression vector | GE Healthcare |
| pMM104 | pET19b/ His-FlhA$_{C}$ | 1 |
| pMMHA001 | pET19b/ His-FlhA$_{C38K}$ | 11 |
| pMM406 | pTrc99A/ His-FliJ | 1 |
| pMKGK2 | pTrc99A/ FlgK | 12 |
| pMMGN101 | pGEX-6p-1/ GST-FlgN | 8 |
| pMMGN130 | pET15b/ His-FlgN | 13 |
| pMMGN140 | pTrc99AFF4/ FlgN | 13 |
| pMMJ1001 | pGEX-6p-1/ GST-FliJ | 14 |
| pMMJ1002 | pGEX-6p-1/ GST-FliJ(Δ13–24) | 5 |

## Supplementary references


1. Minamino, T. & Macnab, R. M. Interactions among components of the *Salmonella* flagellar export apparatus and its substrates. *Mol. Microbiol.* **35**, 1052–1064 (2000).
2. Evans, L.D.B., Stafford, G.P., Ahmed, S., Fraser, G.M. & Hughes, C. An escort mechanism for cycling of export chaperones during flagellum assembly. *Proc. Natl. Acad. Sci. USA* **103**, 17474–17479 (2006).
3. González-Pedrajo, B., Minamino, T., Kihara, M. & Namba, K. Interactions between C ring proteins and export apparatus components: a possible mechanism for facilitating type III protein export. *Mol. Microbiol.* **60**, 984–998 (2006).
4. Bange, G. *et al*. FlhA provides the adaptor for coordinated delivery of late flagella building blocks to the type III secretion system. *Proc. Natl. Acad. Sci. USA* **107**, 11295–11300 (2010).
5. Minamino, T., Morimoto, Y.V., Hara, N. & Namba, K. An energy transduction mechanism used in bacterial type III protein export. *Nat. Commun.* **2**, 475 (2011).
6. Yamaguchi, S., Fujita, H., Sugata, K., Taira, T. & Iino, T. Genetic analysis of *H2*, the structural gene for phase-2 flagellin in *Salmonella*. *J. Gen. Microbiol.* **130**, 255–265 (1984).
7. Ohnishi, K., Ohto, Y., Aizawa, S.-I., Macnab, R.M. & Iino, T. FlgD is a scaffolding protein needed for flagellar hook assembly in *Salmonella typhimurium. J. Bacteriol.* **176**, 2272–2281 (1994).
8. Minamino, T. *et al.* Interaction of a bacterial flagellar chaperone FlgN with FlhA is required for efficient export of its cognate substrates. *Mol Microbiol* **83:** 775–788 (2012).
9. Inoue, Y., Morimoto, Y. V., Namba, K. & Minamino, T. Novel insights into the mechanism of well-ordered assembly of bacterial flagellar proteins in *Salmonella*. *Sci. Rep.* **8**, 1787 (2018).
10. Ohnishi, K., Fan, F., Schoenhals, G.J., Kihara, M. & Macnab R.M. The FliO, FliP, FliQ, and FliR proteins of *Salmonella typhimurium*: putative components for flagellar assembly. *J. Bacteriol.* **179**, 6092–6099 (1997).
11. Saijo-Hamano, Y., Minamino, T., Macnab, R.M. & Namba, K. Structural and functional analysis of the C-terminal cytoplasmic domain of FlhA, an integral membrane component of the type III flagellar protein export apparatus in *Salmonella*. *J. Mol. Biol.* **343**, 457-466 (2004).
12. Furukawa, Y. *et al.* Interactions between bacterial flagellar axial proteins in their monomeric state in solution. *J. Mol. Biol.* **318**, 889–900 (2002).
13. Kinoshita, M. *et al.* Rearrangements of α-helical structures of FlgN chaperone control the binding affinity for its cognate substrates during flagellar type III export. *Mol. Microbiol.* **101**, 656–670 (2016).
14. Minamino, T. *et al*. Role of the C-terminal cytoplasmic domain of FlhA in bacterial flagellar type III protein export. *J. Bacteriol.* **192**, 1929–1936 (2010).
